# ‘They use the little information they have to pass judgement’: A framework analysis of type 1 diabetes stigma experienced by UK adults living with type 1 diabetes

**DOI:** 10.1111/dme.70191

**Published:** 2025-12-13

**Authors:** Harriet Housby, Akaal Kaur, Thomas Wylie, Nick Oliver, Katrina Scior, Vicky McKechnie

**Affiliations:** ^1^ Department of Metabolism, Digestion and Reproduction Imperial College London London UK; ^2^ Research Department of Clinical, Educational and Health Psychology University College London London UK

**Keywords:** social stigma, stigma, type 1 diabetes

## Abstract

**Aims:**

Type 1 diabetes stigma (T1D‐stigma), the negative social judgements towards people living with type 1 diabetes (T1D), is linked to poor psychological well‐being and suboptimal diabetes self‐management. This qualitative study explored the T1D‐stigma experiences of adults living with T1D in the UK.

**Methods:**

UK‐based adults (aged ≥18 years) with T1D completed an online survey as part of a wider T1D‐stigma study. Respondents who endorsed at least one item on the Type 1 diabetes stigma assessment scale (DSAS‐1) were then invited to provide more information about their stigma experiences. Responses were analysed using framework analysis.

**Results:**

The majority of respondents (96%) endorsed experiencing at least one instance of perceived or experienced stigma. A framework analysis of *N* = 337 participants' responses developed five themes and 19 sub‐themes. The first and second themes explored the ‘*Sources*’ and ‘*Drivers and facilitators*’ of T1D‐stigma. The third theme encapsulated the ‘*Experiences*’ of T1D‐stigma, where participants described ‘unsolicited comments and advice’, ‘discrimination and rejection’ and ‘lack of understanding’. The fourth theme encompassed the ‘*Impact*’ of T1D‐stigma, where participants described its social, emotional, and behavioural impact. The final theme explored *Stigma management*’ and described ‘dealing with T1D‐stigma’, ‘changes over time in self, society and diabetes technology’ and ‘protective factors in T1D‐stigma encounters’.

**Conclusion:**

This study has provided the first systematic qualitative analysis of T1D‐stigma in the UK. In line with other diabetes‐related stigma research, T1D‐stigma was broad in its manifestations and impact. Participants volunteered information about T1D‐stigma management, despite not being explicitly asked about this, highlighting approaches that could inform future interventions.

**What's new?**

**What is already known?**

- Type 1 Diabetes Stigma (T1D‐stigma) impacts psychosocial well‐being and diabetes management for people living with type 1 diabetes (T1D).

**What has this study found?**

- Using a framework analysis, this study provides a conceptualisation of the sources, drivers, facilitators, experiences, and impact of T1D‐stigma from the perspective of UK adults. Participants also mentioned ways of managing T1D‐stigma, which warrants further investigation.

**What are the implications of the study?**

- The findings strengthen the evidence and reinforce existing frameworks for T1D‐stigma. Future studies should investigate T1D‐stigma management strategies to learn effective ways of preventing and addressing T1D‐stigma.

## 1 INTRODUCTION

Type 1 diabetes stigma (T1D‐stigma) refers to the negative social judgement of someone living with type 1 diabetes (T1D) that leads to experienced or perceived rejection, exclusion, stereotyping, blame, and/or status loss.^1^, ^2^, ^3^ There is a rapidly growing evidence base demonstrating the burden of navigating T1D‐stigma.^4^ Stigmatising comments about, for example, diet and insulin injections,^5^ can significantly impact psychological and social well‐being, complicating diabetes management and contributing to increased health risks.^6^, ^7^, ^8^, ^9^ Qualitative research in Australia has shown that people with T1D often encounter negative stereotypes and misconceptions about their condition, leading to feelings of shame and isolation.^10^ In the United States, national surveys using patient‐reported outcome measures (PROMs) have documented frequent stigma encounters for people with T1D.^11^ Similar findings have been reported in Switzerland, where individuals with T1D experience significant stigma that impacts their quality of life.^12^

A framework highlighting the causes, experiences, and consequences of diabetes‐related stigma identified mitigating strategies from the broader health stigma research to enhance coping and resilience.^7^ The framework was based on a systematic review of type 1 and type 2 diabetes stigma literature and was underpinned by the conceptualisation of stigma as a social process consisting of labelling, stereotyping, separation, status loss, and discrimination that co‐occur in the context of a group that holds power, stigmatising a group without power.^2^ At the time of the review, there was a paucity of research, and studies lacked standardised measures of diabetes‐related stigma, meaning that the conceptualisation of stigma differed between studies.

Since then, the type 1 Diabetes Stigma Assessment scale (DSAS‐1)^13^ has been developed and validated, and developments in the health stigma field, such as the Health Stigma and Discrimination Framework (HSDF)^14^ have moved away from a ‘stigmatised‐stigmatiser’ distinction seen in previous stigma models^2^ and emphasise that stigma is perpetuated at multiple levels, from individual behaviours to societal systems and structures. According to the HSDF, it is crucial to understand the specific drivers and manifestations of stigma to develop effective interventions, yet comprehensive studies concerning the sources, experiences, and impact of T1D‐stigma in the UK are lacking.^15^

This study aims to address this gap in the literature by exploring the lived experiences of stigma, specifically among adults with T1D in the UK, by using framework analysis of responses to an open‐text question based on the DSAS‐1 about respondents' experiences of T1D‐stigma. This qualitative approach aims to elucidate the sources, drivers, facilitators, experiences, and impact of T1D‐stigma, which is a necessary first step in guiding effective development of targeted interventions and support systems, ultimately enhancing the psychosocial well‐being of individuals living with T1D in the UK.^14^

## 2 METHODS

### 2.1 Study design

The data for this study were extracted from the type 1 Diabetes Stigma Study, a cross‐sectional national survey conducted between August 2024 and April 2025, of the stigma experiences of adults living with T1D in the UK. The protocol and quantitative results of this study are presented in (Paper co‐submitted to Diabetic Medicine^16^).

### 2.2 Procedure

Participants were recruited through three primary pathways. *N* = 3754 were contacted through After Diabetes Diagnosis REsearch Support System (ADDRESS‐2). ADDRESS‐2 is a national study, active since 2011, that individuals newly diagnosed with T1D can join to receive information about relevant research opportunities. *N* = 463 were contacted from a list of individuals who consented to be contacted for future T1D‐related research opportunities at Imperial College London. Lastly, the study used social media advertisements tailored in collaboration with Egality Health, which is a community engagement agency aiming to create health equity by working to engage groups historically under‐represented in research.

The online survey, hosted by Qualtrics™, included a plain English information sheet, a series of eligibility screening questions, followed by a consent form and the survey. Participants could alternatively complete the survey via post and a prepaid return envelope.

### 2.3 Participants

Eligible participants were adults (aged ≥18 years) living with T1D in the UK who were able to complete the survey in English online or via post. In addition, inclusion in the present sub‐study required the completion of a single qualitative free‐text question (see Measures) that appeared for participants who responded ‘agree’ or ‘strongly agree’ to at least one item on the DSAS‐1.

### 2.4 Measures

#### 2.4.1 Type 1 Diabetes Stigma Assessment Scale‐1 (DSAS‐1)

The DSAS‐1^13^ is a 19‐item scale assessing perceived and experienced stigma in individuals with T1D across three subscales: Blame and Judgement (six items), Identity Concerns (seven items), and Treated Differently (six items). Items are rated on a 5‐point Likert scale (1 = Strongly Disagree to 5 = Strongly Agree). Higher scores indicate greater perceived or experienced stigma encounters. Total scores range from 19–95; subscale ranges are 6–30 (Blame and Judgement), 7–35 (Identity Concerns), and 6–30 (Treated Differently). Each subscale has high internal consistency (Cronbach's α = 0.88–0.89).^13^

Participants who responded ‘Agree’ (4) or ‘Strongly Agree’ (5) to at least one item were presented with a single open‐ended question. The question was phrased: ‘For questions where you have ticked “agree” or “strongly agree”, can you tell us more about the different range of experiences, beliefs or actions that you experience as particularly stigmatising? For example, what have you experienced or observed? How did this affect you? Did it change how you view things or yourself, the things you do or used to do, or your relationships?’ There was no character limit for the response, and it was not compulsory to complete this question.

Demographic, clinical characteristics, and well‐being measures were self‐reported by participants.

### 2.5 Data analysis

Qualitative data were screened to identify non‐meaningful responses, including one‐word or short responses, which were not interpretable in reference to the question (e.g. ‘N/A’, ‘nothing’, ‘No’, ‘don't remember the questions’). Consequently, 2.3% of the responses (8/345) were excluded from qualitative analysis. Relevant responses were coded in QSR NVIVO (version 14).

Responses to the question were analysed using framework analysis^17^ to thematically analyse the data.^18^ Two researchers with clinical health psychology and qualitative research experience (VM and HH) were involved in the iterative response coding and review process, which broadly followed six steps: familiarisation, coding, developing a working analytical framework, applying the framework, charting the data into the framework matrix and interpreting the data. The first step of framework analysis, transcription, was not required as the data were collected via an online survey.

HH familiarised themselves with all open text responses and coded the first 100, creating 83 base codes and grouped the codes to create a working analytic framework. Following this, VM read the first 100 responses for orientation and then reviewed the working analytic framework. The working framework was discussed by VM and HH, and revisions were made accordingly to arrive at an agreed framework with 74 base codes. Following this, for the first 100 responses, VM reviewed all coded sections of text under each base code and recorded where she had a question or disagreement. In total, 602 coded sections of text were reviewed, and 42 queries were raised (7.0% of coded sections). Queries or disagreements were discussed and resolved in the following ways: renaming an existing code to better reflect the responses within it; creating an additional base code; re‐coding or uncoding the data; leaving the code as it originally was. Through discussion, full agreement was reached. Following this, HH re‐coded the first 100 responses as required, then coded the remaining responses within the agreed final framework using NVivo. As this study analysed a large number of open‐text responses to the survey, it was impractical and not meaningful to chart the data from each respondent as outlined in the original methodology paper; however, as the coding took place in NVivo, the authors were able to view participant quotes ‘charted’ under the relevant codes, sub‐themes, and overarching themes to view and select illustrative quotes. Finally, patterns and relationships between themes were interpreted and discussed. All authors reviewed the final framework and interpretations to ensure that it represented the data adequately and reflected the research question.

Quantitative data were uploaded and analysed with SPSS Statistics (version 24; IBM). Descriptive statistics were calculated for demographic and clinical characteristics of eligible participants (i.e., those with a meaningful response). In addition, differences between those eligible participants who provided a response (meaningful or otherwise) and non‐respondents were assessed using Mann–Whitney U and Pearson's Chi‐squared test for continuous and categorical data, respectively.

### 2.6 Positionality

The analysis was led by HH, who is a clinical psychology research assistant with qualitative research experience in physical and mental health settings. It was supported and supervised by VM, a T1D specialist clinical research psychologist. HH and VM met regularly to discuss the analysis and development of themes and sub‐themes, as well as to consider how their positions may influence the interpretation of findings.

### 2.7 Ethics

The Imperial College Research Ethics Committee approved the Type 1 Diabetes Stigma study (reference number: 6986692).

## 3 RESULTS

In total, *N* = 438 completed the DSAS‐1. The mean total score was 59/95 (SD 13.4). Mean scores for the three subscales were as follows: blame and judgement 22.4/35 (SD 4.9); identity concerns 21.5/30 (SD 6.4); treated differently 15.1/30 (SD 5.0).

Of those who completed the DSAS‐1, *N* = 420 (96%) endorsed at least one item and were shown the open‐text question. *N* = 345 (82% of those endorsing one item) replied to the single qualitative free‐text question, with 337 (98% of respondents) providing meaningful responses. Of those who provided meaningful responses, the median word count was 63 (IQR = 31.0–111.0).

Demographic and clinical characteristics of respondents (*N* = 345) and non‐respondents (*N* = 75) were mostly equivalent, except that respondents were more likely to be women than non‐respondents (*n* = 221, 64.1% vs. *n* = 37, 49.3%; *p* = 0.018).

### 3.1 Participant characteristics

Table 1 summarises the demographic and clinical characteristics of participants who provided meaningful responses (*N* = 337). The median age was 41 (30.0–54.0), the median duration of diabetes was 10 years, and 47% used an insulin pump.

**TABLE 1:** Sociodemographic, clinical, and psychosocial characteristics of participants who gave meaningful responses (*N* = 337).

**Participant characteristics**		
Sociodemographic characteristics		
Women *N* (%)	219	(65)
Age, years (*Mdn*, *IQR*)	41.0	(30.0–54.0)
Ethnicity^a^ *N* (%)		
White	313	(93)
Mixed	10	(3.0)
Black	9	(2.7)
Other	5	(1.5)
Religion (*N*, %)		
No religion	131	(39)
Christian	123	(37)
Atheist	41	(12)
Agnostic	17	(5.0)
Prefer not to say	14	(4.1)
Other	11	(3.3)
Education (*N*, %)		
Postgraduate degree	123	(37)
Undergraduate degree	120	(36)
A‐level	54	(16)
GCSE or equivalent	29	(8.6)
Apprenticeship	11	(3.3)
IMD (*Mdn*, *IQR*)	7.0	(4.0–9.0)
**Clinical characteristics**		
Age diagnosed with diabetes (years) (*Mdn*, *IQR*)	24.0	(12.0–37.0)
Diabetes duration (years) (*Mdn*, *IQR*)	10.0	(6.0–24.7)
HbA1c (mmol/mol) (*Mdn*, *IQR*)	52	(46–58)
BMI (*Mdn*, *IQR*)	25.0	(22.5–28.7)
Time in range (%) (*Mdn*, *IQR*)	73	(60–84)
Number of hypos in the last week (*Mdn*, *IQR*)	3.0	(1.0–4.7)
Number of severe hypos in last year (*Mdn*, *IQR*)	0.0	(0.0)
Insulin delivery (*N*, %)		
Multiple daily injections	175	(52)
Insulin pump with CGM linkage	118	(35)
Insulin pump	44	(13)
Glucose monitoring (*N*, %)		
Continuous glucose monitoring	318	(94)
Self‐monitoring of blood glucose	19	(5.6)
Diabetes course attendance (*N*, %)	206	(61)
Retinal screening past year (*N*, %)	324	(96)
Urine sample past year (*N*, %)	280	(83)
Foot check past year (*N*, %)	274	(81)
**Psychosocial characteristics**		
Diabetes Impact (DIDP; 0–49) (*Mdn*, *IQR*)	34.0	32–37
Anxiety (GAD‐7; 0–21) (*Mdn*, *IQR*)	5.0	3–9
Depression (PHQ‐9; 0–24) (*Mdn*, *IQR*)	5.0	3–10
Diabetes stigma (M, SD)		
DSAS‐1 – total score (19–95)	59	13.4
Blame and judgement (7–35)	22.4	4.9
Identity concerns (6–30)	21.5	6.4
Treated differently (6–30)	15.1	5.0

^a^ Using the UK government's agreed list of ethnic groups.

### 3.2 Themes

**FIGURE 1:**
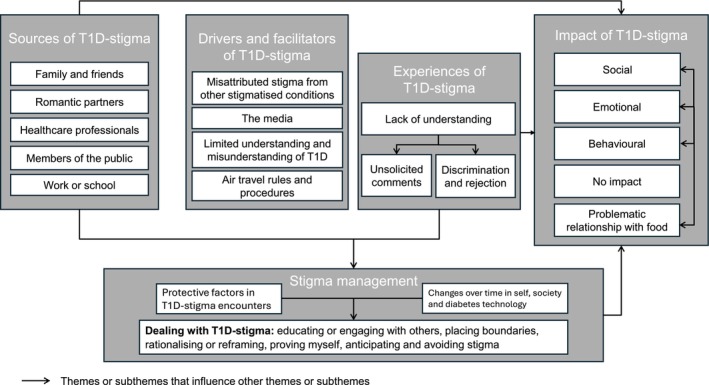
A framework for understanding T1D‐stigma according to participants' experiences, with arrows indicating influential relationships between different themes and sub‐themes.

The framework analysis identified five themes and 19 sub‐themes to represent the range of lived experiences of T1D‐stigma in response to the open‐text question. Each theme is supported with relevant extracts from the open text responses. Figure 1 depicts a framework for understanding T1D‐stigma according to participants' experiences and relationships between different themes. Table 2 lists all themes and sub‐themes, with example quotation(s) from respondents.

**TABLE 2:** Themes and sub‐themes, with number of participants endorsing each theme or sub‐theme, a description and example quotation(s) from respondents.

Theme and sub‐theme	Number endorsing the theme and sub‐theme	Sub‐theme description	Illustrative quotation
Sources	128		
Family and Friends	33	Stigmatising comments or actions from friends and family	‘*Because I haven't had diabetes since birth, I find weirdly that people who've known me for a long time are the most resistant*’. (*P490*)
Romantic partners	17	Stigmatising comments or actions from romantic partners	‘*I remember an ex‐boyfriend when I was 20 telling me he could never marry someone with diabetes*’. (*P686*)
Healthcare professionals	27	Stigma from healthcare professionals, both non‐specialist and specialist	‘*I have had care teams make disparaging or patronising remarks about my management*’ (*P853*)
Members of the public	24	Stigmatising encounters with members of the public	‘*I've had strangers make fun of my insulin pump*’ (*P051*)
Work or school	64	Stigmatising comments or actions from at work or school	‘*I have struggled to work day and night shifts in the same week due to struggling to change basal rates*, etc. *When i have tried to discuss this with employers it has been dismissed, i have been seen as causing trouble or trying to avoid particular shift patterns*’. (*P322*)
Drivers and Facilitators	111		
Limited understanding and misunderstanding of T1D	57	Comments about limited understanding and misunderstanding of T1D, driving T1D‐stigma	‘*People have said* ‘*oh that means you can't have sugar if you have diabetes*’ *or they use the little information they have to pass judgement*’. (*P033*)
Misattributed stigma from other stigmatised conditions or behaviours	68	Stigma relating to type 2 diabetes, obesity, or illicit drug use is driving T1D stigma	‘*I think that sometimes people are unaware of the differences between type 1 and 2 diabetes and make judgements based on their limited knowledge of both, for example, that “I can*'*t eat any sugar at all”, and will make a comment if they see/or hear me mention a sugary food*’. (*P148*) ‘*I was injecting in a McDonald*'*s and an older couple complained to the management that I was “taking heroin”. I was 12 years old!*’ (*P120*)
The media	7	Instances of T1D stigma being perpetuated by the media	‘*And on TV with comics using diabetes as a lazy shortcut joke for the size of a cake or a meal*’ (*P327*)
Air travel rules and procedures	2		‘*air cabin stewards don*'*t understand that hypos can be fixed simply in a short time and can prevent you boarding if your sugar is too low*’ (*P862*)
Experiences	244		
Lack of Understanding	66	Others do not understand what is involved in type 1 Diabetes management Others conflate type 1 and type 2 diabetes	‘*From my experience, people often get type 1 and type 2 diabetes confused. They believe they are the same condition…. I know that individuals without diabetes do not fully understand how much is involved in managing type 1 diabetes*’. (*P033*)
Unsolicited comments	201	Negative reactions to diabetes equipment Blame for diagnosis and day‐to‐day management Assumptions about what people can and cannot do	‘*You have diabetes, you look so healthy!*’ ‘*you swam that far? You have diabetes! How did you do it?*’ ‘*Of course you can do shift work, other people with diabetes manage to do shift work fine!*’ (*P020*)
Discrimination and rejection	65	Being excluded from opportunities Discrimination at work or school Loss of personal relationships due to T1D‐stigma	‘*[employer] were not willing/understanding that I need time off work to attend appointments. I have also been reprimanded when I have taken too much time of sick*’ (*P067*) ‘*[On a date] He was very concerned, said he couldn*'*t cope with “medical issues” and I never heard from him again*’. (*P474*)
Impact	158		
Behavioural	62	Behaviours as a consequence of T1D‐stigma encounters: avoiding disclosing diagnosis, avoiding doing what I want, changing or delaying management, pushing myself, or justifying behaviours	‘*I have continued to live life at a faster pace and do more just to prove there is no limit to having this condition*’. (*P028*)
Social	33	Social impact following T1D‐stigma encounter, for example, strained existing relationships or anxiety when meeting new people	‘*I have had rows with my husband who has blamed me for hypos, and or exhaustion due to diabetes*’. (*P527*) ‘*more awkward when I meet new people because I don*'*t know how they are going to react or act towards me*’. (*P362*)
Emotional	118	Emotional impact following T1D‐stigma encounter. Includes internalising emotions for example, increased anxiety, self‐consciousness and depression, and externalising emotions such as anger and frustration	‘*It made me very insecure, I questioned my own instincts, and I felt completely ashamed*’. (*P068*) ‘*Comments from my closest friends and family made me feel more isolated and afraid*’. (*P215*) ‘*Constantly challenged if I eat something sweet. Made me angry. I was told that I shouldn*'*t inject at the table in a restaurant. Made me angry*’. (*P355*)
No impact	4	Participants clearly state that T1D‐stigma encounters have no impact on their lives	‘*I think people are surprised by the fact that I can eat pretty much whatever I want, whenever I want. I don*'*t think they react negatively to this but it is definitely a stigma. However, I don*'*t feel that it changes what I do and it doesn*'*t affect me*’. (*P222*)
Relationship with food	2	Negative impact on the relationship with food that cuts across behavioural, emotional social and social impact. For example, disordered eating habits	‘*I remember one vivid time at school where a teacher embarrassed me by asking my why I was eating in class while I had a mouthful of my snack and had to reply and after then I never eat another snack again. At other times, I have experienced extremely inadequate and at times negligent “care” for my diabetes within eating disorder hospital units and was always viewed as “too complex” to treat or made to feel like a huge hassle*’. (*P323*)
Stigma Management	170		
Dealing with T1D‐stigma	139	Dealing with T1D‐stigma includes: educating or engaging with others placing boundaries rationalising or reframing proving myself anticipating and avoiding stigma	‘*As I got older, I learned that my health was more important than how people felt about my condition, and I began to start taking better care of myself, and now I rarely ever feel embarrassed by sharing that I*'*m a diabetic*’. (*P059*) ‘*I fear that if people see me injecting my insulin in the open especially at work or in social situations such as a restaurant, I would feel that they are all looking at me. I would rather go into the toilet and take my insulin which I do*’. (*P761*)
Change over time in self, society and diabetes technology	37	Defined as: changes in the individual change in society change in diabetes technology and the impact this has on T1D‐stigma management	‘*I am lucky that I am a lot stronger and more confident in my diabetes management now, but if some of the things that happened to me would have happen in the months after my diagnosis, it would have been devastating*’. (*P798*) ‘*I used to challenge people and try to offer some education about it, but it became so frequent and tiring I*'*d just say thank you and move on*’ (*P850*) ‘*It is worth noting that the stigma has lessened in recent years… I think there is greater public knowledge about CGM and CSII now, so the comments and rude reactions are far less frequent*’. (*P322*) ‘*Switching from MDI to CSII both reduced my embarrassment in terms of injecting in public but also increased it in terms of having it attached to my body, especially when I am being intimate*’. (*P397*)
Protective factors in T1D stigma encounters	50	Factors that ‘protect’ participants from the negative impact of T1DS such as: Seeing prominent figures with T1D Peer and community support Feeling positive about diabetes Having a personal trait or identity that impacts how stigma is managed	‘*These experiences don*'*t change my self‐view because my Mum is a T1D, I was diagnosed at a young age, and Mum instilled in me a secure identity around the diagnosis*’. (*P574*) ‘*I have actually been fortunate in that I am quite a go‐getter so try to make sure that I almost do more to show them that Type 1 diabetes doesn*'*t have to stop me*’. (*P671*)

#### 3.2.1 Theme 1 | Sources of T1D‐stigma

Participants named where and from whom they had experienced T1D‐stigma. This included family and friends, romantic partners, healthcare professionals (including diabetes specialists), members of the public, and at school or work.

#### 3.2.2 Theme 2 | Drivers and facilitators of T1D‐stigma

Participants also described what they understood to be driving, maintaining, or facilitating T1D‐stigma. This included limited understanding or misunderstanding of T1D and misattributed stigma from other stigmatised conditions or behaviours, in particular type 2 diabetes, obesity, and illicit drug use. Airline travel rules and procedures and the media were also experienced as facilitating stigma by showing poor or stigmatising examples of treatment of people with diabetes and perpetuating misunderstanding and misattributed stigma:

The media's lack of distinction between T1 and T2, or explainers of the disease, perpetuates this misunderstanding, which has massive psychological impacts on disease sufferers. I also strongly believe that it has an impact on health care settings and misunderstanding of the seriousness for care (P724)

#### 3.2.3 Theme 3 | Experiences of T1D‐stigma

Whilst some participants reported that T1D‐stigma encounters were infrequent, ‘*I think the only stigma I've ever encountered is the occasional comment if I'm eating something sugary…I've never experienced anyone treat me any worse because of diabetes*’ (P242), others provided rich detail about one or more stigmatising experiences. There were three sub‐themes: lack of understanding, unsolicited comments, and discrimination and rejection.

##### Lack of understanding

Participants stated that others often conflated type 1 and type 2 diabetes, expressing frustration and a desire to distance the two conditions, with some participants suggesting that renaming the conditions would be helpful. Further, they reported that people did not understand what was involved in managing T1D, in particular that delaying management could have serious consequences, and that T1D management was a full‐time and exhausting job.

##### Unsolicited comments

Participants described negative reactions to their diabetes equipment, including stares or comments when injecting, negative comments about sensors or pumps, and others thinking something was wrong when they attended to their diabetes. Participants also reported experiences of blame for diagnosis and management, including comments that T1D was caused by their actions in childhood and sometimes that it was still present due to their actions today, or blame or judgement about day‐to‐day management. Finally, they reported that others made assumptions about what they could and could not do, including comments about food choices, activities, and not having the appearance of someone who has diabetes.

##### Discrimination and rejection

Participants described being excluded from opportunities such as holidays or social events because friends were worried about them having a hypo, being discriminated against in the workplace or at school; for example, colleagues and teachers preventing them from engaging in physical activities or being reprimanded for treating hypos. People also described losing personal relationships due to T1D‐stigma; for example, romantic partners taking a step back due to fear that T1D would be a burden and/or may impact future children. Some experiences of unsolicited comments, discrimination, and rejection were perceived to be related to a lack of understanding of T1D; however, others were in spite of the individual's perceived understanding of T1D:

Even those who do have a greater understanding can become frustrated with it: yet another episode of hypoglycaemia becomes something for which one is blamed. (P036)

#### 3.2.4 Theme 4 | Impact of T1D‐stigma

Participants shared that T1D‐stigma had a broad impact across socio‐emotional and behavioural aspects of their lives.

##### Social

Some participants reported that T1D‐stigma put a strain on existing relationships with friends, family, or romantic partners. Participants also reported anxiety about meeting new people in anticipation of T1D‐stigma.

##### Emotional

Following T1D‐stigma, participants reported experiencing internalising emotions such as anxiety, depression, self‐consciousness, lack of self‐confidence, embarrassment, guilt, shame, and feeling a burden on others, and externalising emotions like frustration and anger.

##### Behavioural

Participants reported that T1D‐stigma led them to avoid disclosing their diagnosis, avoid doing what they wanted to do (such as eating, avoiding certain foods, or going to social events that involve exercise), change or delay management, justify their actions, or push themselves hard to prove others wrong.

##### Problematic relationship with food

Some participants also found that T1D‐stigma negatively impacted their relationship with food due to judgement around diet, which in turn impacted social, emotional, and behavioural aspects of their life.

##### No impact

Others, despite endorsing at least one item on the DSAS‐1, reported that T1D‐stigma had little or no impact.

Emotional, social, and behavioural impacts of T1D‐stigma were closely interlinked. For example, one participant cited that the impact of bullying led to them developing a negative self‐view which ‘caused’ them to change day‐to‐day T1D management:

It made me feel embarrassed and stressed with my illness I was bullied when I was diagnosed because of my diabetes, so I had a negative view of myself and diabetes from that. This caused me to not look after myself properly, like not taking my insulin and carrying on with exercise when I was low (P931)

All three aspects of the emotional, social, and behavioural impact of T1D‐stigma appeared to be embodied where participants reported a consequent problematic relationship with food.

Impact appeared to be influenced by the source of stigma; for example, T1D‐stigma encounters seemed to be particularly difficult when coming from those with a ‘greater understanding’, such as doctors, employers, family, and friends:

The people who have had the reactions I describe are medical doctors, lawyers, accountants, bankers, fund managers, and CEOs. This reaction has ultimately led to a strained relationship with my family, unhealthy workplace practices (e.g., not disclosing my condition) and poor health management. (P036)

#### 3.2.5 Theme 5 | T1D‐stigma management

Despite not being explicitly asked about stigma management, participants shared ways that they managed stigma, and this theme encapsulates their management approaches, some of which were to protect them from being negatively impacted by T1D‐stigma: ‘*dealing with T1D‐stigma*’, ‘*changes over time in self, society and diabetes technology*’, and ‘*protective factors in T1D‐stigma encounters*’.

##### Dealing with T1D‐stigma

Participants wrote about ‘engaging and educating others’, including making complaints or taking legal action, correcting misconceptions, and teaching others about the impact of the condition. Participants also talked about ‘placing boundaries’, for example, that their experiences with T1D‐stigma had led them to distance or end relationships, take a step back from work, avoid disclosing or showing their diabetes, and prioritising their needs over those of strangers. Other participants seemed to use ‘rationalising or reframing’ as a coping strategy for T1D‐stigma, for example, that others meant well, were uneducated, or that their situation could be worse. Some participants shared that they felt the need to push or prove themselves in the face of T1D‐stigma to cope with stigmatising encounters. Finally, participants shared that they managed stigma encounters by anticipating them so that they could either avoid or mentally prepare for the encounter.

##### Changes over time in self, society and diabetes technology

Participants shared that their experience of T1D‐stigma management had changed over time, due to changes in themselves, society, and diabetes technology. Of those who mentioned individual change, some said that T1D‐stigma had become easier to deal with, whilst others shared that it was harder to manage, and they felt that they no longer coped with it as they did previously. Those who wrote about society changing felt that there was greater public awareness of T1D and consequently less T1D‐stigma. There were mixed responses concerning T1D technology; some felt it reduced T1D‐stigma, particularly comments around injecting in public, as management was more discreet. However, others noted that it had changed the nature and content of T1D‐stigma; whilst embarrassment around injections was reduced, having a pump attached to the body was stigmatising. Further, one participant shared that whilst technology reduced the time spent thinking about self‐management, it also meant that others underestimated how much time and effort was required, which meant there were more stigma encounters relating to blame and frustrations around management.

##### Protective factors in T1D‐stigma encounters

Some participants described factors that protected them from being negatively impacted by stigmatising encounters. These included seeing prominent public figures with T1D, peer or community support, feeling positively about their diabetes, or a personal trait or identity that impacted how they dealt with stigma.

The theme of stigma management also appeared to relate to the themes of sources of T1D‐stigma, the experience of T1D‐stigma, and the impact of T1D‐stigma. Strategies for dealing with T1D‐stigma differed depending on the source of the T1D‐stigma (known to the person vs. a stranger) and the nature of the experience (unsolicited negative comment vs. lack of understanding). The impact of T1D‐stigma appeared to relate to the presence or absence of protective factors (e.g., a supportive family member) and experiences over time (changes in themselves, society, and diabetes technology).

## 4 DISCUSSION

This framework analysis of T1D‐stigma experienced by adults living with T1D in the UK highlights the broad sources, drivers, experiences, impact, and ways of dealing with T1D‐stigma. It also proposes potential relationships between these factors.

The current framework, discussed in more detail below, reports broadly similar sources, drivers, experiences, and impact of T1D‐stigma to the initial frameworks describing diabetes‐related stigma^7^ and T1D‐stigma.^1^ The study extends these frameworks by summarising participants' ways of dealing with T1D‐stigma encounters, including how these management strategies appear to change over time with changes in self, society, and diabetes technology and in the presence or absence of ‘protective factors’.

Supporting prior literature^4^, ^7^ the perceived sources of T1D‐stigma included romantic partners, family, colleagues, healthcare professionals (HCPs), and members of the public. The finding that not all sources of T1D‐stigma appeared to have the same impact aligns with a study in Australia, which found that participants reported blame, particularly from family members and health care professionals, as more salient.^1^ This suggests that the negative impact of T1D‐stigma may be related to the perceived or expected knowledge of the individual expressing T1D‐stigma and how well they know the person. Language use among healthcare professionals, in particular, has been demonstrated to be particularly problematic^9^ and set as a priority to address by people living with T1D in the UK.^15^ Training recommended by the American Diabetes Association to healthcare staff on weight bias and stigma^19^ may be helpfully adapted to target reducing T1D‐stigma amongst HCPs in the UK.

The sub‐theme of misunderstanding and misattributed stigma as drivers of T1D‐stigma supports previous findings relating to diabetes stigma.^4^, ^7^ Participants reported experiencing stigma related to other conditions or stigmatised behaviours such as illicit substance use or type 2 diabetes, suggesting that T1D‐stigma is, in part, comprised of misattributed stigma from other highly stigmatised conditions or behaviours. Previous work found that people living with T1D experience being called a ‘druggie’ and expressed frustration at having stereotypes associated with type 2 diabetes, such as eating sugar or being overweight, applied to them.^1^ This suggests that interventions targeted at increasing awareness and understanding of other stigmatised conditions and behaviours may also be beneficial.

The finding that the media seemed to be facilitating T1D‐stigma and a driver, or maintaining factor, through setting an example of poor treatment and perpetuating misunderstanding and misattributed stigma, accords with previous findings^1^, ^20^ suggesting that interventions that involve altering media messaging may be particularly helpful in preventing T1D‐stigma.

The third theme, capturing participants' lived experiences of T1D‐stigma: unsolicited comments and advice, lack of understanding, and discrimination and rejection, is consistent with experiences reported in the broader diabetes stigma literature.^4^, ^21^ A key frustration of participants was that others used the little information they had about T1D to make assumptions and pass judgement. In particular, participants frequently reported others conflating T1D with type 2 diabetes, and consequently experiencing people's stigmatising attitudes about type 2 diabetes. This was perceived to contribute to stigmatising assumptions and comments and could explain some participants' desire to rename T1D to distinguish it more clearly from type 2 and reduce associated stigma, which has been documented in prior research.^1^

Participants' descriptions of the socio‐emotional and behavioural impact of T1D‐stigma align with findings from prior reviews^8^, ^22^ which highlight the association between T1D‐stigma and internalising emotions such as guilt and shame, withdrawal behaviours, and delays in self‐management, underscoring the importance of addressing stigma to improve well‐being. However, not all participants who experienced T1D‐stigma reported being negatively impacted by it; this is important to acknowledge, as it suggests that endorsing items on the DSAS‐1 does not necessarily imply that individuals are negatively impacted by such encounters. It also highlights that more work needs to be done to understand the differential impact of T1D‐stigma and identify effective mitigating strategies for different subgroups.

Indeed, although not explicitly asked, participants shared how they managed T1D‐stigma encounters, which has provided some insight into understanding its differential impact. For example, the finding that stigma management strategies change within participants over time follows the concept of developmental stages; as people age, they generally become less concerned with others' opinions, though it could also relate to changes in the individual's relationship with their diabetes over time.^23^ Participants also reported that T1D‐stigma has lessened in recent years, as knowledge on a societal level has improved. This supports the aims of the #EndDiabetesStigma campaign, an international consensus and pledge launched in 2024 to end diabetes stigma^4^ and suggests that interventions focused on raising awareness of T1D may continue to help address and prevent T1D‐stigma.^4^

The mixed reports about the advancement in diabetes equipment influencing T1D‐stigma both within and between participants appear to reflect the literature around the individual's conflicting relationship with diabetes technology.^24^ Switching from multiple daily injections to an insulin pump reduces the misattributed stigma associated with needle use. However, the visibility of pumps and sensors can make individuals living with T1D more self‐conscious, as whilst the technology may be more socially acceptable than a needle, it is also more permanent in the sense that it is fixed to the body.^24^, ^25^ Further, the technology appears to be perceived as decreasing others' awareness of the relentless daily task of managing T1D and increasing others' frustrations and blame around the daily reality of fluctuating glucose and hypoglycaemic and hyperglycaemic episodes.

Similar to previous research, for example, about gestational diabetes stigma,^26^ the presence of ‘protective factors in T1D‐stigma encounters’ such as peer support, a particular identity or personality trait, or how participants were feeling about their diabetes, appeared to influence how participants experienced and were impacted by T1D‐stigma encounters. Such insights are important in considerations around the development of diabetes stigma interventions and in supporting individuals affected by T1D‐stigma. Additionally, the finding that strategies for dealing with T1D‐stigma differed depending on the source of the T1D‐stigma and the nature of the experience suggests there is likely an interplay between these dynamics that determines how someone experiences or is impacted by T1D‐stigma that warrants further investigation. Qualitative research that allows for more in‐depth consideration of this would be useful.

### 4.1 Strengths and limitations

This study provides the first systematic qualitative analysis of T1D‐stigma using endorsement of an item on a standardised measure, the DSAS‐1, in the UK. The majority of respondents (96%) endorsed experiencing at least one instance of perceived or experienced stigma, with over three‐quarters of respondents providing relatively detailed accounts of their experiences. This suggests it is a topic that is meaningful to people living with T1D.^15^ Responses from 337 participants allowed for analysis of a broad range of responses, which included an understanding of how frequently certain experiences were reported. The method of data collection and number of responses did not, however, allow for in‐depth analysis of individuals' experiences. Future qualitative research should aim to address this by gaining a more in‐depth understanding of people's experiences.

In line with previous literature, respondents to both the open‐text question and the survey overall were more likely to be women.^27^ In spite of efforts to target people from groups historically under‐represented in research, a high percentage of respondents were White. Insulin pump use was also higher than in the general T1D population. So, it would be helpful to hear more specifically about the T1D‐stigma experiences of men and people who use multiple daily injections to administer insulin, and those from minoritised ethnicities, to see if and how they differ.

### 4.2 Future direction

Findings from this study suggest that interventions targeted at raising awareness of T1D, in particular addressing misconceptions around T1D, may be helpful in preventing T1D‐stigma encounters in the UK. However, the finding that T1D stigma appears to include an amalgamation of misattributed stigma from other stigmatised conditions and behaviours is worth investigating further, as T1D‐stigma interventions targeted at increasing knowledge alone may not be effective in reducing stigmatising encounters, for example, unsolicited comments about insulin injections, which may be motivated by needle use stigma. Further, the finding that not all sources and experiences of T1D‐stigma appeared to have the same impact warrants future investigation, as understanding further about T1D‐stigma encounters that are particularly negatively impactful may help to identify effective strategies for preventing T1D‐stigma encounters.

Whilst it is not the responsibility of people living withT1D to address T1D‐stigma, the diabetes stigma consensus highlights the importance of co‐creating stigma interventions, such as multilevel interventions recommended through participatory action research conducted in Iran.^28^ Further qualitative investigation of people's experiences of stigma management in the UK could be a similarly helpful next step in informing areas for effective T1D‐stigma intervention development and prevention strategies.

## FUNDING INFORMATION

This study was funded by Diabetes UK (Grant no: 23/0006506)

## CONFLICT OF INTEREST STATEMENT

None to declare.

## Data Availability

The data that support the findings of this study are available on request from the corresponding author. The data are not publicly available due to privacy or ethical restrictions.
